# The Competitive Orthopaedic Trauma Fellowship Applicant: A Program Director's Perspective

**DOI:** 10.5435/JAAOSGlobal-D-20-00175

**Published:** 2021-05-06

**Authors:** M. Kareem Shaath, Stephen J. Warner, James F. Kellam, Timothy S. Achor

**Affiliations:** From the Orlando Health Jewett Orthopedic Institute, Florida State College of Medicine, University of Central Florida College of Medicine, Orlando, FL (Dr. Shaath), and the Division of Orthopaedic Trauma, Department of Orthopaedic Surgery, McGovern Medical School at UTHealth, Houston, TX (Dr. Warner, Dr. Kellam, and Dr. Achor).

## Abstract

**Introduction:**

In 2018, orthopaedic trauma had the lowest match rate among orthopaedic subspecialties. The purpose of this study was to determine the importance of factors evaluated by orthopaedic trauma fellowship directors when ranking applicants after the interview.

**Methods:**

An electronic survey was submitted to fellowship directors and consisted of 16 factors included in a fellowship application. Respondents were asked to rate the importance of these factors for applicants they interviewed on a 1 to 5 Likert scale, with 1 being not at all important and 5 being critical.

**Results:**

Thirty-seven fellowship directors responded (63.8%). The highest-rated factor was the applicant interview (mean score 4.82), followed by the quality of letters of recommendation (4.69), personal connections made to the applicant (3.89), and potential to be leader (3.86). Fellowship directors at academic programs rated interest in an academic career (*P* = 0.003), research experience (*P* = 0.023), and exposure to well-known orthopaedic traumatologists (*P* = 0.003) higher than their counterparts at private institutions. Programs with more than one fellow rated potential to be a leader higher than programs with one fellow (*P* = 0.02).

**Discussion:**

Trainees may use this study when compiling an application to optimize their chances of matching at the program of their choice.

Orthopaedic residency programs are intended to provide residents a well-rounded exposure to all aspects of the field. However, increasing pressure for subspecialization and duty hour restrictions has developed a heightened interest in trainees pursuing subspecialty training within orthopaedic surgery. A recent study has shown that up to 91% of orthopaedic residency graduates intend to complete additional subspecialty training, whereas a growing number of graduating residents are planning on completing multiple postgraduation fellowships.^1,2,3,4^

Orthopaedic trauma is a subspecialty demonstrating an increased interest by orthopaedic residents, with nearly 20% of residents applying for a trauma fellowship position each year.^5^ From 2015 to 2018, there was an increase from 71 applicants to 104 applicants, whereas the number of positions offered increased from 78 to 86.^5,6^ Predictably, with the increase in applicants, the match rate decreased from 86% to 78% (Figure 1).^5,6^ For comparison, the available 2017 match rates for other orthopaedic subspecialties are as follows: spine 83%, foot and ankle 87%, shoulder and elbow 88%, pediatrics 89%, hand 90%, and sports medicine 97%.^5,6^ Because orthopaedic trauma has been the most competitive subspecialty for matching into a fellowship position over the past decade, the importance of a high-quality fellowship application is further emphasized.^5,6^

**Figure 1:**
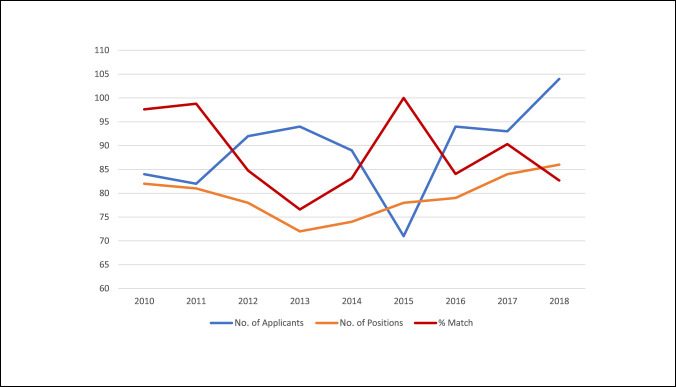
Graph demonstrating the number of applicants (blue line), the number of positions offered (orange line), and the match rate (red line) by year.

The fellowship application for orthopaedic trauma has many components, which may be used to evaluate applicants such as the curriculum vitae, letters of recommendation, research experience, and the interview. The relative importance of these components in determining the selection of a fellow remains unclear because no study has evaluated the factors involved in the ranking of applicants. The purpose of this study was to determine the relative importance of these components to orthopaedic trauma fellowship directors when ranking applicants. We hypothesized that orthopaedic trauma fellowship directors will prioritize certain components of the application when ranking their applicants. We believe that this information will be valuable to trainees planning careers in orthopaedic trauma given the increasing competition for securing a fellowship position in this field.

## Methods

**Figure 2:**
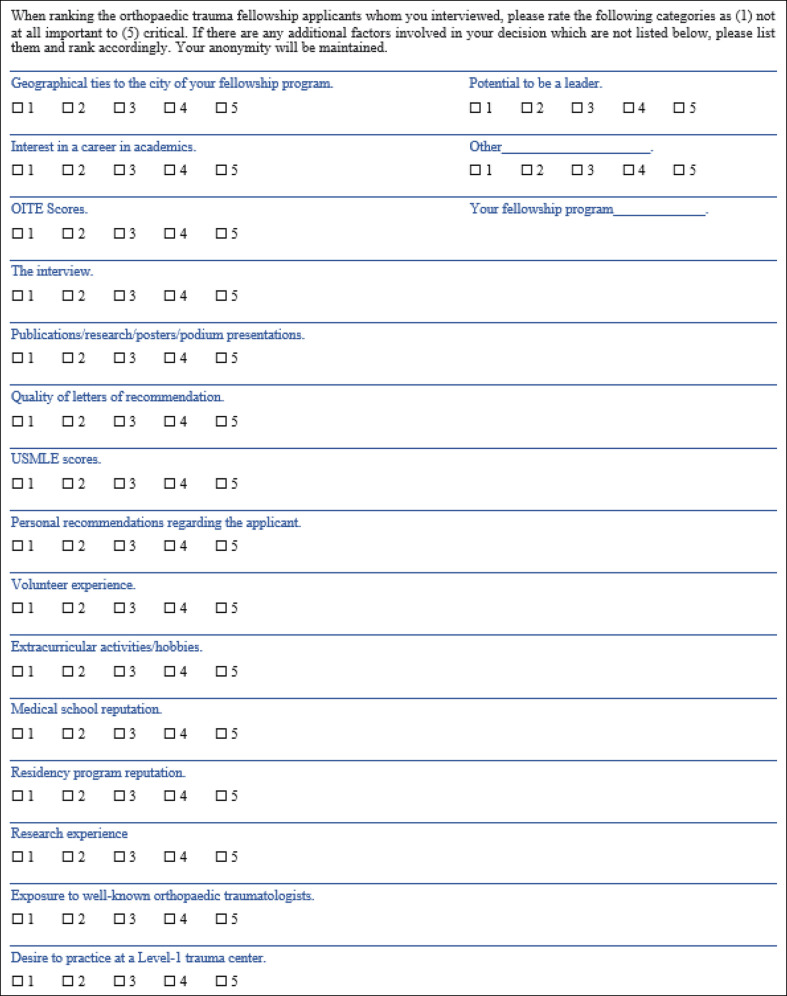
Image showing the survey that was distributed to fellowship directors.

A complete list of orthopaedic trauma fellowships and fellowship director e-mails was obtained from the website of the Orthopaedic Trauma Association (OTA).^7^ Of the 59 programs listed, 9 are accredited by the Accreditation Council for Graduate Medical Education and the remaining 48 are accredited by the OTA. The senior author, TSA, is a fellowship director and his participation from the study was excluded, leaving 58 fellowship directors eligible for participation. An electronic survey, based on a previously validated survey, was submitted to all 58 fellowship directors by e-mail using Google Forms (Mountain View, CA)^8^ (Figure 2). The survey was modified and consisted of a list of 16 factors included in the process for applying to orthopaedic trauma fellowship. The respondents were asked to rate the importance of these factors for applicants they interviewed. The question order was randomized, and all items were ranked on a 1 to 5 Likert scale, with 1 being not at all important and 5 being critical. The senior author contacted nonresponders through e-mail to encourage their participation. The scores for each factor were analyzed by calculating the mean Likert score and SD for each item surveyed. A two-sample *t*-test was used to analyze the data between two groups, and a one-way analysis of variance was used when comparing more than two groups, with a *P* value less than 0.05 considered significant.

## Results

Of the 58 fellowship directors eligible for participation, 37 responded, a response rate of 63.8%. There were 23 programs with one fellow and 14 programs with greater than one fellow. Thirty programs were affiliated with residency programs, whereas 7 were community-based. Three programs were affiliated with level-2 trauma centers, whereas the rest were level-1 trauma centers (Table 1).

**Table 1:** Description of Programs

Category	No. of Programs
No. of fellows	
1 fellow	23
>1 fellow	14
Residency affiliation	
No affiliation	7
Affiliation with a residency	30
Trauma center designation	
Level 1	34
Level 2	3
Geography	
East coast	7
Midwest	14
South	8
West coast	8
Accreditation	
OTA	31
ACGME	6

ACGME = Accreditation Council for Graduate Medical Education, OTA = Orthopaedic Trauma Association

**Figure 3:**
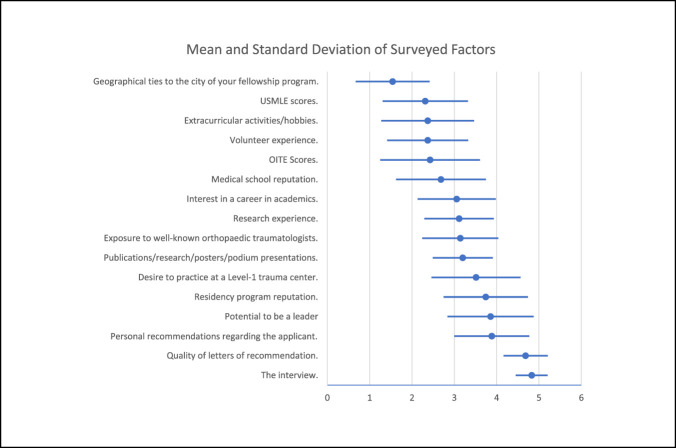
Graph demonstrating mean (blue circles) and the SD range (blue bars) of rated factors.

Of the 16 factors listed on the survey, the most important factor was the applicant interview (mean 4.82, SD 0.38). This was followed by the quality of letters of recommendation (4.69, 0.52), personal recommendations regarding the applicant (3.89, 0.89), potential to be a leader (3.86, 1.02), and the reputation of the residency program of the applicant (3.79, 1.0). The three lowest-rated factors were extracurricular activities/hobbies (2.37, 1.10), United States Medical Licensing Examination (USMLE) scores (2.31, 1.00), and geographical ties to the city of the fellowship program (1.54, 0.87). The complete data set is illustrated in Figure 3. When comparing the mean ratings of the interview and the quality of letters of recommendation, fellowship directors feel that they are more important than personal recommendations and all other factors surveyed (4.86 compared with 3.95 [95% confidence interval (CI) 0.61-1.28]; *P* < 0.001) and (4.67 compared with 3.95 [95% CI 0.45-1.16]; *P* < 0.0001).

Additional analysis was performed to determine whether different fellowship settings and characteristics influenced responses. Fellowship directors at academic programs rated several factors higher when compared with their counterparts at private institutions. These factors were an interest in an academic career (3.39 compared with 1.86 [95% CI 0.68-2.37]; *P* = 0.003), research experience (3.86 compared with 2.29 [95% CI 0.20-2.0]; *P* = 0.023), and exposure to well-known orthopaedic traumatologists during residency (3.46 compared with 2.20 [95% CI 0.68-2.24]; *P* = 0.003). Programs with greater than one fellow rated the potential to be a leader higher than their counterparts at programs with only one fellow (4.31 compared with 3.59 [95% CI 0.11-1.33]; *P* = 0.02). No significant differences were observed when analyzing programs by geographic location or Accreditation Council for Graduate Medical Education versus OTA accreditation.

## Discussion

Our data demonstrate the differential relative importance of certain factors in the applications for orthopaedic trauma fellowship, as determined by fellowship directors. Uniformly, they find the interview to be the most important factor followed by quality of the letters of recommendation and personal recommendations regarding the applicant. Geographical ties to the program, USMLE scores, volunteer experience, and Orthopaedic In-Training Examination scores were much less important, however. This information is critical for trainees when prioritizing their efforts and drafting their application for securing a competitive fellowship position in orthopaedic trauma.

Multiple studies in other subspecialties similarly found that the interview is the most important factor in ranking an applicant.^8–10^ Grabowski and Walker,^11^ in a survey of various orthopaedic fellowship directors across all subspecialties, also found that the interview was the most important factor in ranking an applicant. The interview was also found to be the most important factor when ranking medical students for orthopaedic surgery residency positions and was found to have the highest correlation with the final rank of the applicant.^12^ Although the applicant may have the opportunity to interact with fellowship directors during informational sessions, courses, or site visits, the interview is the only formal time through the application process that the applicant has direct interaction with their potential fellowship program and can demonstrate their communication skills, maturity level, self-confidence, the ability to listen and articulate thoughts, and personality fit within the program.^10^

Standardized tests have been shown to be very important in the selection of orthopaedic residents.^13^ Given the low ratings given to Orthopaedic In-Training Examination and USMLE scores, it is our opinion that orthopaedic trauma fellowship directors do not feel that a standardized, multiple-choice examination is predictive of success within the field of orthopaedic trauma. This holds true among other orthopaedic subspecialties as well.^8,10,13^

Orthopaedic trauma fellowship directors rated personal recommendations higher than other subspecialties.^8,10,13^ In a similar survey, orthopaedic sports medicine fellowship directors rated personal recommendations as the fifth most important factor, whereas we found it to be the third most important factor surveyed.^8^ Personal recommendations may hold greater importance in orthopaedic traumatology because it is a much smaller network than sports medicine. It has been suggested that when applicants have positive personal connections with a program either directly or through their mentors, this increases their likelihood to match at that particular fellowship program.^8^

Another interesting finding was that publications and research experience were the seventh and ninth important factors ranked by fellowship directors, respectively. It should be noted, however, that academic programs did rank research experience markedly higher than their nonacademic counterparts. A previous study found that subspecialty research was one of the five most important factors in obtaining an interview to an orthopaedic fellowship program.^11^ In that study, participation in research, irrespective of authorship, was found to be important by 90% of respondents and nearly 5% of fellowship directors felt that the applicant needed to be first author.^11^ Our study suggests that orthopaedic trauma fellowship directors feel that there are more important factors than research, although most fellowship programs have a research requirement of at least one paper per year.^14^

We found that fellowship directors at programs affiliated with academic institutions rated certain factors higher than their counterparts at programs that were not affiliated with an academic institution. This is a finding that has not been demonstrated in previous studies. We found that fellowship directors at academic programs placed higher value on an interest in pursuing an academic career, research experience, and exposure to well-known traumatologists during residency.

The major strength of our study is the response rate. A response rate of 63.8% is considered excellent for an electronic survey.^15^ The limitations to our study are that of many survey studies. The surveys were not submitted anonymously, and this may have influenced participation in the study and the responses. In using the averages of Likert scores, we ordered the importance of items based on these averages. It is possible that our results may have been different if the respondents were asked to directly rank the topics presented. In addition, the number of items surveyed was based on a previously validated study but was not exhaustive.^8^ However, we did allow fellowship directors to enter free-text responses for any other factors we did not include on the survey, but no consistent themes were available and only one program director inserted a comment.

This is the first study to evaluate factors that orthopaedic trauma fellowship directors consider when ranking applicants. With the increasing numbers of applicants and competition for orthopaedic trauma fellowship positions, we believe that our study provides those interested in pursuing a career in orthopaedic trauma with useful information.

Based on the results of this study, the ideal candidate for an academic trauma fellowship will have research experience, have had exposure to orthopaedic traumatologists during residency, and have an interest in an academic career. They must also have excellent letters of recommendation and demonstrate the potential to be a leader during the interview. Candidates for nonacademic orthopaedic trauma fellowships do not need extensive research experience and may come from a program that does not have prominent faculty. However, they must also interview well and have excellent letters of recommendation.

Based on our results, residents who wish to subspecialize in orthopaedic trauma should build a relationship with orthopaedic trauma surgeons during their residency as this may assist them in securing a fellowship position as the surgeon may make a personal recommendation on their behalf. Overall, we believe that trainees may use this study to assist with compiling a strong application to optimize their chances of matching at the program of their choice in the increasingly competitive field of orthopaedic trauma.
